# Enhanced timing abilities in percussionists generalize to rhythms without a musical beat

**DOI:** 10.3389/fnhum.2014.01003

**Published:** 2014-12-10

**Authors:** Daniel J. Cameron, Jessica A. Grahn

**Affiliations:** ^1^Brain and Mind Institute, University of Western OntarioLondon, ON, Canada; ^2^Department of Psychology, University of Western OntarioLondon, ON, Canada

**Keywords:** rhythm perception, expertise, entrainment, beat perception, tapping

## Abstract

The ability to entrain movements to music is arguably universal, but it is unclear how specialized training may influence this. Previous research suggests that percussionists have superior temporal precision in perception and production tasks. Such superiority may be limited to temporal sequences that resemble real music or, alternatively, may generalize to musically implausible sequences. To test this, percussionists and nonpercussionists completed two tasks that used rhythmic sequences varying in musical plausibility. In the beat tapping task, participants tapped with the beat of a rhythmic sequence over 3 stages: finding the beat (as an initial sequence played), continuation of the beat (as a second sequence was introduced and played simultaneously), and switching to a second beat (the initial sequence finished, leaving only the second). The meters of the two sequences were either congruent or incongruent, as were their tempi (minimum inter-onset intervals). In the rhythm reproduction task, participants reproduced rhythms of four types, ranging from high to low musical plausibility: Metric simple rhythms induced a strong sense of the beat, metric complex rhythms induced a weaker sense of the beat, nonmetric rhythms had no beat, and jittered nonmetric rhythms also had no beat as well as low temporal predictability. For both tasks, percussionists performed more accurately than nonpercussionists. In addition, both groups were better with musically plausible than implausible conditions. Overall, the percussionists' superior abilities to entrain to, and reproduce, rhythms generalized to musically implausible sequences.

## Introduction

The ability to entrain one's movements to the beat in musical rhythm appears to be a human universal (Drake and Bertrand, 2001). Entrainment of neural activity to the beat may underlie this behavior (Snyder and Large, 2005; Will and Berg, 2007; Fujioka et al., 2009, 2012; Nozaradan et al., 2011, 2012, 2013). Although beat-related behavior and neural entrainment occur in humans generally, musicians have better perception and production of rhythm, and synchronization to the beat, than nonmusicians (Drake, 1993; Drake et al., 2000). Musicians also have different patterns of neural activations in response to rhythms compared to nonmusicians (Grahn and Brett, 2007; Chen et al., 2008; Grahn and Rowe, 2009). Percussionists are particularly likely to show enhanced rhythmic abilities compared to other musicians, due to the rhythmic focus of their training. However, because the context of their expertise is music (and usually only beat-based music), percussionists' measureable advantages in rhythmic abilities may not generalize to all types of temporal sequences, only those that are musically plausible and have a beat. Different neural substrates underlie beat-based (or relative) timing and nonbeat-based (or absolute) timing (Teki et al., 2011). Because musical rhythms are beat-based, the beat-based timing mechanism may be selectively affected by musical training. However, because musical rhythm likely relies on both relative and absolute timing (the absolute time interval between notes is important, as well as the time of those notes relative to the beat), training may tune both relative and absolute timing mechanisms. Thus, we expect percussionists to have greater abilities in both beat-based and nonbeat-based contexts.

Percussionists' enhanced rhythm and timing abilities have been previously examined. Percussionists maintain more veridical reproduction of single time intervals than both nonpercussionist musicians and nonmusicians (whose reproductions are more biased by the statistical distribution of the set of intervals they are tested on) (Cicchini et al., 2012). Drummers are better at perceiving audio-visual asynchrony in point-light videos of drumming movement (Petrini et al., 2009, 2010). Therefore, percussionists have superior abilities in some temporal tasks. However, it is not known if their superiority extends beyond single intervals to sequences of intervals and if so, whether it extends only to musically plausible sequences, or to all types of sequences. It seems likely that percussionists would have superior performance with sequences that are musically rhythmic, but it is less clear that they would have superior performance with sequences that do not have a beat structure and are thus musically implausible. In support of this, Repp (2005) reports the case of a percussionist who excelled in tapping on the beat, but performed at only an average level when tapping at (less musically salient) off-beat positions. Although only a single case, it suggests that enhanced ability to produce rhythms may be limited to rhythms that have a beat structure, and are thus musically plausible. In another study, expert percussionists outperformed novice musicians in synchronized tapping tasks (Fischinger, 2009), but their precision was reduced when simultaneously performing a distracting task (Fischinger, 2011), demonstrating that expert tapping is subject to limiting factors.

Percussionists who train with some types of contemporary music are likely to have experience perceiving and producing rhythms that are irregular (e.g., rhythms with odd subdivisions of the beat, such as groups of 5 or 7, and different consecutive subdivisions). However, these rhythms are still likely to be notated by the composer, and subsequently represented by the performer, relative to a metrical, beat-based structure, though this may not be perceivable by the listener.

To better understand the extent and nature of percussionists' superior rhythm perception and production performance, we tested how expert percussionists performed with rhythms that had a strong or weak beat structure. We used two tasks to examine percussionists' and nonpercussionists' rhythm abilities: a beat tapping task, and a rhythm reproduction task. For each task we manipulated the beat structure of the rhythms, and thus, musical plausibility.

In the beat tapping task, we tested percussionists' and nonpercussionists' abilities to accurately tap to the beat of a repeating rhythm across three stages. In the finding stage, a single rhythm was played and participants tapped the beat once they began to perceive it. In the continuation stage, a second, simultaneous rhythm was gradually introduced and the two rhythms played concurrently. In the third stage, called switching, the first rhythm ended, leaving only the second rhythm. After participants began tapping in the finding stage, they carried on tapping through the continuation and switching stages.

We manipulated the tempo and the metrical structure of the rhythmic sequences to create different degrees of beat structure. To manipulate beat structure, the tempi (absolute duration of the basic unit, or minimum inter-onset interval, in the rhythm) of the two rhythms in a trial were either congruent or incongruent. If the two co-occurring rhythms had incongruent tempi, then they would lack a coherent beat structure as an overall rhythmic stimulus. In addition, the metrical structure of rhythms was either triple or duple. Rhythms with a triple metrical structure had 3 basic units per beat, and rhythms with a duple structure had 4 basic units per beat. Thus, the metrical structures of the two co-occurring rhythms were either congruent (both duple or both triple), or incongruent (one duple and one triple).

**Figure 1:**
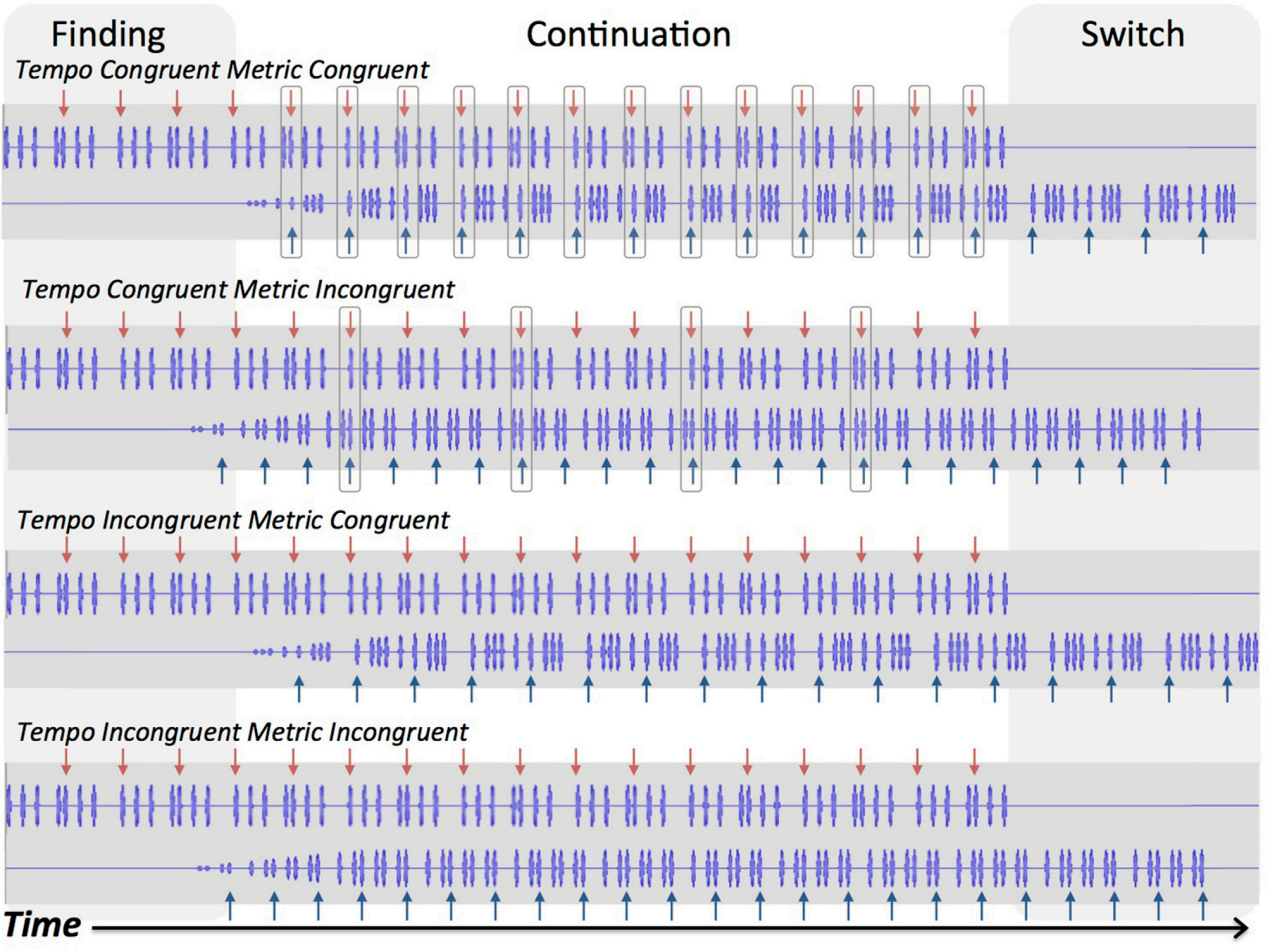
**Waveforms of rhythmic sequences**. Arrows indicate beat positions for each sequence. Boxed positions indicate coinciding beat positions. Shaded areas indicate the finding and switching stages of trials, and the white area indicates continuation.

We consider tempo incongruence to eliminate beat structure because the intervals between tones in one sequence are no longer integer ratios of the interval between tones in the other sequence. The two sequences use tones of the same pitch, so they are integrated into a single stream of noninteger ratio intervals. No commonly heard or played music contains simultaneous rhythms played at slightly different tempi, and for this reason we consider tempo incongruent conditions to be musically implausible. As can be seen in Figure 1, in the tempo incongruent conditions, the beat never aligns between the two sequences, regardless of metrical congruence. In contrast, metrical incongruence preserves beat structure, because the underlying pulse, or basic unit, is common to both sequences. Thus, the intervals in one sequence are integer ratios of the intervals in the other sequence, and the second, introduced, sequence can be incorporated into the metrical structure of the initial rhythm. Although metrical incongruence is somewhat uncommon in Western music, it is musically plausible (assuming there is tempo congruence; e.g., “cross rhythm,” polyrhythm, or polymeter). Figure 1 shows how tempo and metric congruence affect alignment of beats and basic units of the two sequences in each of the four conditions. The beat positions in both sequences always align in tempo congruent metric congruent trials, and only sometimes align in tempo congruent metric incongruent trials. Thus, the tempo congruent metric congruent trials have alignment of basic units (tempo congruence), and of beats (metric congruence), whereas the tempo congruent metric incongruent trials have alignment of units but only some alignment of beats. In contrast, the tempo *in*congruent conditions (metric congruent and incongruent) have neither alignment of basic units nor of beats. For these reasons, tempo incongruence is more rhythmically challenging and less musically plausible than metrical incongruence.

We predicted that variability and accuracy of beat tapping would be worse for both percussionists and nonpercussionists in the tempo incongruent conditions. However, if percussionists' superior abilities in tapping the beat generalize to nonbeat, musically implausible contexts, then their tapping should be less variable and more accurate than nonpercussionists' in all conditions (tempo incongruent and tempo congruent), regardless of beat structure. Conversely, if percussionists' superior abilities are limited only to beat-based, musically plausible conditions, then they should perform better than nonpercussionists only for tempo congruent trials.

We predicted that, in tempo congruent conditions, metrical incongruence of the sequences would not influence beat tapping because the common duration of the basic unit would allow integration of the second rhythm into the metrical structure of the first. In tempo incongruent conditions, the lack of a common underlying pulse rendered the metrical congruence irrelevant. In the switching stage, we expected that metrical incongruence and tempo incongruence would impair beat tapping, as any incongruence would force participants to adjust their tapping to the new beat rate when the first sequence ended.

In the rhythm reproduction task, we tested percussionists' and nonpercussionists' timing accuracy by testing their ability to accurately reproduce rhythms. The rhythms were of four types: metric simple, metric complex, nonmetric (similar to Grahn and Brett, 2007), and jittered nonmetric (similar to Grahn and Schuit, 2012). These types correspond to having (in order) a strong beat structure, a weak beat structure, no beat structure, and no beat structure as well as low temporal predictability (a greater number of distinct interval lengths were used in jittered nonmetric rhythms than in the other rhythms). We expected that, for both percussionists and nonpercussionists, rhythm reproduction accuracy would decrease as beat structure weakened across the 4 conditions. For the two conditions with no beat structure, we predicted that reproduction accuracy would be lower in the jittered nonmetric condition than the nonmetric condition. If percussionists' enhanced rhythm abilities depend on the beat, then their superior rhythm reproduction performance should be limited to those conditions with a strong—and possibly those with a weak—beat structure, and should not extend to the two conditions with no beat. Conversely, if their enhanced abilities extend beyond beat-based, musically plausible rhythms, then percussionists should outperform nonpercussionists on all types of rhythms, regardless of structure.

## Materials and methods

### Participants

25 percussionists (6 female; 23.9 mean years of age; 12.6 mean years of formal musical training; 8.8 mean years of percussion lessons; 13.5 mean years of playing percussion) were recruited from the faculties of music at the University of Toronto (Canada) and Western University (London, ON, Canada). Most were undergraduate students studying percussion, but our sample also included graduate students, professionals, and one faculty member. All percussionists practiced regularly at the time of testing and reported a mean of 15.4 h of playing percussion per week. Thirty nonpercussionists (11 female; mean age 21.0 years; 7.4 years of music training) were recruited from London, ON, Canada. Although many nonpercussionists had musical training, only 13 were actively practicing (mean 4.2 h per week), and none had studied music at the university level or played professionally.

### Stimuli

#### Beat tapping task

For the beat tapping task, 4 types of trials were used, each composed of two rhythmic tone sequences (see Figure 1). Each individual sequence contained repetitions of a beat-based rhythm composed of 375 Hz sine tones lasting 100 ms, and amplitude ramped up/down over the first/final 50 ms of each tone. All rhythms had a structure of 4 metrical beats with either 4 or 3 basic units (corresponding to meters of 4/4 or 12/8, in musical terminology). In each trial, the two sequences were presented in a staggered fashion. The two sequences had equal length of either 6 or 8 repetitions of the basic rhythmic pattern, depending on the meter (6 repetitions for 4/4 sequences, 8 repetitions for 12/8 sequences). The first sequence began, and the second sequence was gradually introduced (the amplitude ramped up linearly over the course of its first iteration of the rhythm). From the onset of the first sequence, the second sequence was introduced after a period equal to two repetitions of its basic rhythm. Both patterns played together at equal intensity, and after the first rhythm ended, the second sequence continued. The staggered structure gave 3 distinct stages: finding (during which only the first sequence was heard), continuation (during which the second sequence was introduced and both sequences were heard together), and switching (during which the second sequence was heard alone).

For each trial, sequences were paired such that their meters and tempi were congruent (same) or incongruent (different). Thus, four types of trials were possible: tempo congruent meter congruent (TCMC), tempo congruent meter incongruent (TCMI), tempo incongruent meter congruent (TIMC), and tempo incongruent meter incongruent (TIMI). All sequences were initially constructed using one of three tempi, having a minimum inter-onset interval of 180, 210, or 245 ms. For tempo incongruent trials, one sequence had one of these tempi, and the other had a slightly slower one (minimum inter-onset interval of 185, 215.83, or 251.81 ms, respectively).

#### Rhythm reproduction task

**Figure 2:**
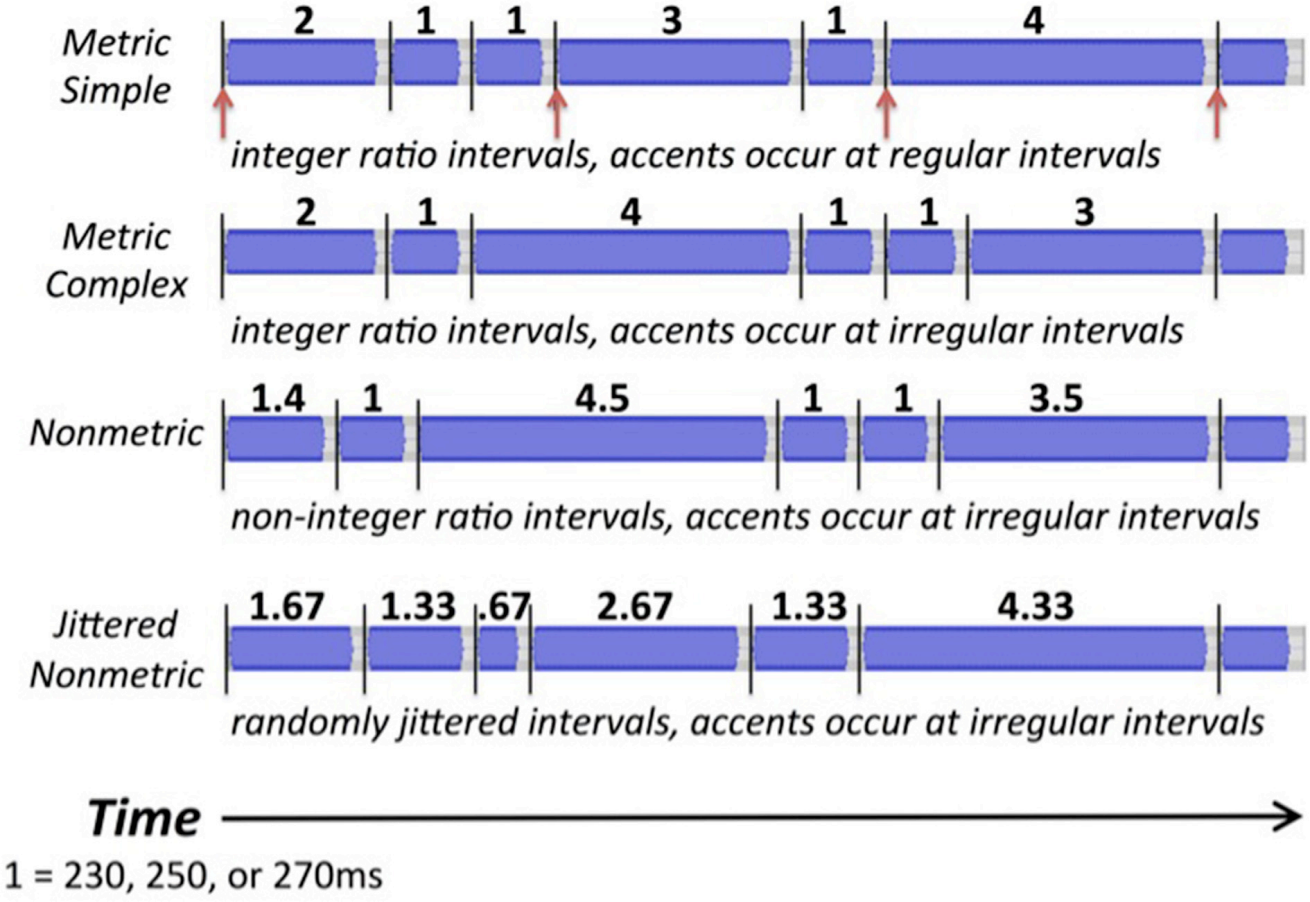
**Waveforms of the four stimulus types for the reproduction task: metric simple, metric complex, nonmetric, and jittered nonmetric**. Black lines highlight onsets. Red arrows indicate beat positions in the metric simple condition.

For the reproduction task, 4 types of rhythmic tone sequences were used (see Figure 2). Metric simple (MS) rhythms consisted of intervals that were whole integer ratios of one another (e.g., relative durations of 1, 2, 3, or 4), and a tone always occurred on the four metrical beat positions. Metric complex (MC) rhythms also consisted of intervals that were whole integer ratios, but did not have events occurring on each metrical beat position. Nonmetric (NM) rhythms consisted of intervals that were not whole integer ratios of one another (relative durations of 1, 1.4, 3.5, 4.5), and thus had no beat structure. These three rhythm types are similar to those used and described in previous studies (Grahn and Brett, 2007). Jittered Nonmetric (JNM) rhythms consisted of intervals that also were not integer ratios. The JNM rhythms were created by pseudorandomly increasing or decreasing the intervals from metric simple rhythms by a fixed percentage of the tempo, and leaving some intervals unchanged (creating relative durations of 0.67, 1, 1.33, 1.67, 2, 2.33, 2.67, 3, 3.33, 3.67, 4, 4.33). This alteration resulted in sequences that were inherently less predictable than the other conditions, because as a group they consisted of a larger set of interval durations (12 possible interval durations for each tempo) than the other conditions (4 possible interval durations for each tempo). The jittered nonmetric rhythms have been used previously (Grahn and Schuit, 2012). For all conditions, the tones in each rhythm were filled sine tones lasting the length of the inter-onset interval minus 40 ms, creating a silent gap between each tone. Each tone's amplitude was uniform aside from being ramped up and down over the first and final 10 ms. Trials varied in tempo, having a relative duration of 1 equal to 230, 250, or 270 ms. The entire set of stimulus sequences is shown in Table 1.

**Table 1:** **Rhythmic sequences used in the reproduction task**.

**Condition**	**Interval structure**	**Condition**	**Interval structure**
MS	1 1 2 4 2 2	NM	1 1 1 1.4 4.5 1 1.4
MS	2 2 3 1 2 1 1	NM	1.4 3.5 1 4.5 1.4
MS	4 3 1 2 2	NM	4.5 1 1 3.5 1 1 1
MS	1 1 1 1 3 1	NM	1 1 3.5 1 1 1
MS	2 2 2 1 1	NM	1.4 1 1.4 1.4 1
MS	2 2 3 1	NM	1.4 4.5 1 1.4
MC	2 3 1 4 2	JNM	0.67 1 1 1.33 2.33 2 3.67
MC	2 4 1 2 2 1	JNM	2.67 1 4.33 1.67 2.33
MC	4 1 1 3 1 2	JNM	3.67 2.33 1.67 0.67 1.33 2.33
MC	1 2 2 1 1 1	JNM	2.33 0.67 1.33 3.67
MC	1 4 1 2	JNM	2.33 1 0.67 0.67 1.33
MC	2 4 2	JNM	2.67 1 2.33 0.67 1.33

Examples of stimulus rhythms of all conditions from both tasks can be accessed in the Supplementary Material.

### Procedure

All participants completed both tasks in a maximum of 35 min, and the order of the two tasks was counterbalanced. For both tasks, participants sat at a desk and used a laptop. Auditory stimuli were presented through noise-canceling headphones. Two thirds of participants in each group tapped on an ErgoDex DX1 response pad and the other third used the laptop keyboard. This was because of equipment limitations (only 2 response pads) and a necessary constraint of testing 3 percussionists simultaneously. Participants could choose which hand to tap with. An experimenter gave verbal instructions, and written instructions were displayed on the laptop.

#### Beat tapping task

For the beat tapping task, participants were instructed to tap along with their perception of the beat as the rhythm unfolded. Participants were instructed that their perception of the beat might change over the course of the trial, and that their tapping might naturally adapt to their perception, but to avoid intentionally changing metrical interpretation or beat rate when not induced to by the stimuli (i.e., to not change when they tapped just to make the tapping more interesting). Trial order was randomized with no trials repeated, and each participant completed six trials of each condition, for a total of 24 trials. Two practice trials were completed before beginning the task.

#### Rhythm reproduction task

For the reproduction task, trials consisted of one presentation of the test rhythm, followed by a visual cue to begin the reproduction attempt. Participants were instructed to reproduce the rhythm as accurately as possible, and were given the criteria for accurate reproduction: the correct number of intervals, with each interval duration within 20% of the presented interval duration. For each trial, participants were given five attempts to accurately reproduce the rhythm. If the reproduction attempt was accurate, they would proceed to the next trial (a new rhythm). If the reproduction attempt was inaccurate, participants would again hear two presentations of the rhythm and would attempt to reproduce it. After five inaccurate attempts for a given trial, they would proceed to the next trial. The accuracy of all reproduction attempts was recorded for analysis. Trial order was randomized with no trials repeated, and there were six trials of each type of rhythm, for a total 24 trials. Before beginning the task, participants completed three practice trials and could repeat the same practice trials again if they were unsure of the task requirements.

### Analyses

#### Beat tapping task

For the beat tapping task, two dependent variables were assessed. The first was coefficient of variation (CV) of inter-tap intervals (ITI). For each trial, the mean and *SD* of the ITIs was calculated. CV was defined as the *SD* of ITIs divided by the mean ITI. The CV of ITIs is the proportional variability of beat tapping.

The second dependent variable was average asynchrony, defined as the average of the absolute differences between beat times and tap times in a sequence. Beat times were determined based on the structure of the tone sequences. As different metrical levels (multiples of the basic temporal unit) of a given rhythm can be correctly identified as “the beat,” each trial's beat positions were determined by the plausible inter-beat interval (IBI) nearest to the participant's mean ITI for that trial. For sequences with four units per beat (4/4 meter), plausible IBIs were 1, 2, 4, 8, and 16 times the tempo, and for sequences with three units per beat (12/8), plausible IBIs were, 1, 2, 3, 4, 6, and 12 times the tempo. This accounted for individual differences in selection of tapping rate with the constraint of comparing taps only to beat positions at rates that were plausible metrical interpretations of the rhythm.

For the finding stage, CVs and asynchronies were each compared between groups using independent samples *t*-tests, as only a single rhythmic sequence was present during finding, and therefore there was no congruence or incongruence of tempo or meter.

For the continuation and switching stages, CV and asynchrony measures were analyzed in a 2 × 2 × 2 × 2 mixed design ANOVA with the within subject factors of tempo congruence (congruent vs. incongruent), metrical congruence (congruent vs. incongruent), and stage (continuation vs. switching), and between subjects factor of group (percussionists vs. nonpercussionists). For each trial, ITIs were determined to be outliers and removed if they were either <0.5 or >1.5 times the mean ITI. This outlier removal procedure was performed once, then the mean ITI was recalculated and the procedure was performed again.

#### Rhythm reproduction task

Reproduction trials were considered accurate if the correct number of intervals was reproduced, and the duration of all reproduced intervals was within 20% of the presented duration. The proportion of rhythms that were accurately reproduced was recorded for each condition, and analyzed in a 2 × 4 mixed design ANOVA with the within subject factor of rhythm type (metric simple, metric complex, nonmetric, and jittered nonmetric), and between subjects factor of group (percussionists vs. nonpercussionists). For both tasks, follow-up independent or paired samples *t-*tests were completed to explore the nature of statistically significant interactions.

## Results

### Beat tapping task

Percussionists had lower CV of ITIs, indicating less variable beat tapping (*CV* = 0.052) compared to nonpercussionists (*CV* = 0.115) during the finding stage [*t*_(50)_ = 4.17, *p* < 0.001]. From the ANOVA considering metrical congruence, tempo congruence, stage, and group, percussionists had less variable beat tapping (*CV* = 0.052) than nonpercussionists (*CV* = 0.127) [main effect of group: *F*_(1, 50)_ = 24.46, *p* < 0.001]. Tapping was more variable when tempi were incongruent [main effect of tempo congruence: *F*_(1, 50)_ = 19.58, *p* < 0.001], and tapping was more variable during the switching period of trials compared to continuation [a main effect of stage: *F*_(1, 50)_ = 5.51, *p* = 0.023].

**Figure 3:**
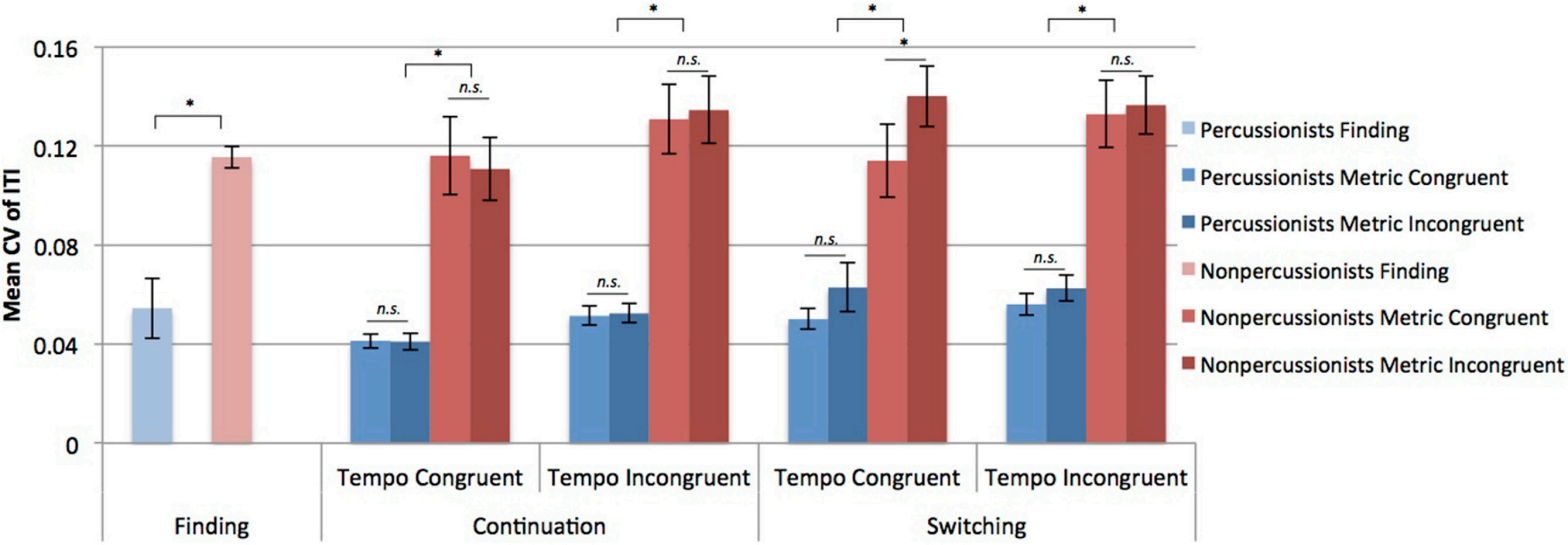
**Mean coefficients of variation (CV) of inter-tap intervals (ITI)**. Error bars indicate ± 1 s.e.m. ^*^Indicates *p* < 0.01 in a paired samples *t*-test.

The effects of tempo incongruence and stage interacted [*F*_(1, 50)_ = 4.84, *p* = 0.033], as did effects of metrical incongruence and stage [*F*_(1, 50)_ = 5.97, *p* = 0.018]. However, all three of the factors also interacted [*F*_(1, 50)_ = 6.62, *p* = 0.013], such that tapping was more variable during TCMI compared to TCMC during switching [*t*_(51)_ = 2.91, *p* = 0.002], but not during continuation [*t*_(51)_ = 0.76, *p* = 0.77], as shown in Figure 3.

The mean absolute asynchrony between the temporal positions of taps and beat positions reflects accuracy of beat tapping. Percussionists had more accurate tapping (lower mean absolute asynchrony) than nonpercussionists during the finding stage of trials [*t*_(50)_ = 7.13, *p* < 0.001]. From the ANOVA, percussionists had greater tapping accuracy (lower asynchrony) than nonpercussionists [main effect of group: *F*_(1, 50)_ = 9.01, *p* = 0.004]. As in the case of CV, tapping was less accurate for tempo incongruent than tempo congruent trials [a main effect of tempo congruence: *F*_(1, 50)_ = 77.04, *p* < 0.001], and less accurate during the switching stage than during continuation [a main effect of stage: *F*_(1, 50)_ = 14.87, *p* < 0.001]. However, unlike CV, tapping was also less accurate for metrically incongruent trials compared to metrically congruent [a main effect of meter congruence: *F*_(1, 50)_ = 14.14, *p* < 0.001].

**Figure 4:**
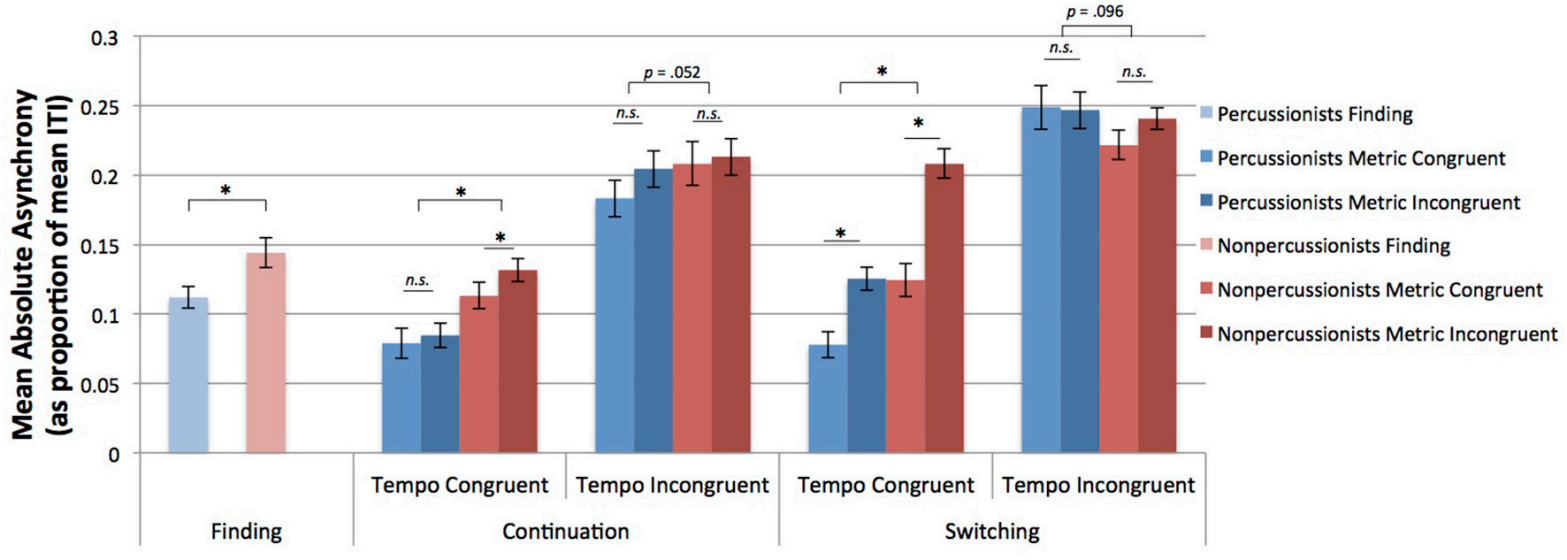
**Mean absolute asynchrony of taps vs. beat positions, as a proportion of the inter-beat interval**. Error bars indicate ± 1 s.e.m. ^*^Indicates *p* < 0.05. Percussionists performed better than nonpercussionists for tempo congruent conditions. However, for tempo incongruent conditions, the difference between groups depended on stage: percussionists tapped more accurately than nonpercussionists during continuation, but nonpercussionists tapped more accurately than percussionists during switching.

**Figure 5:**
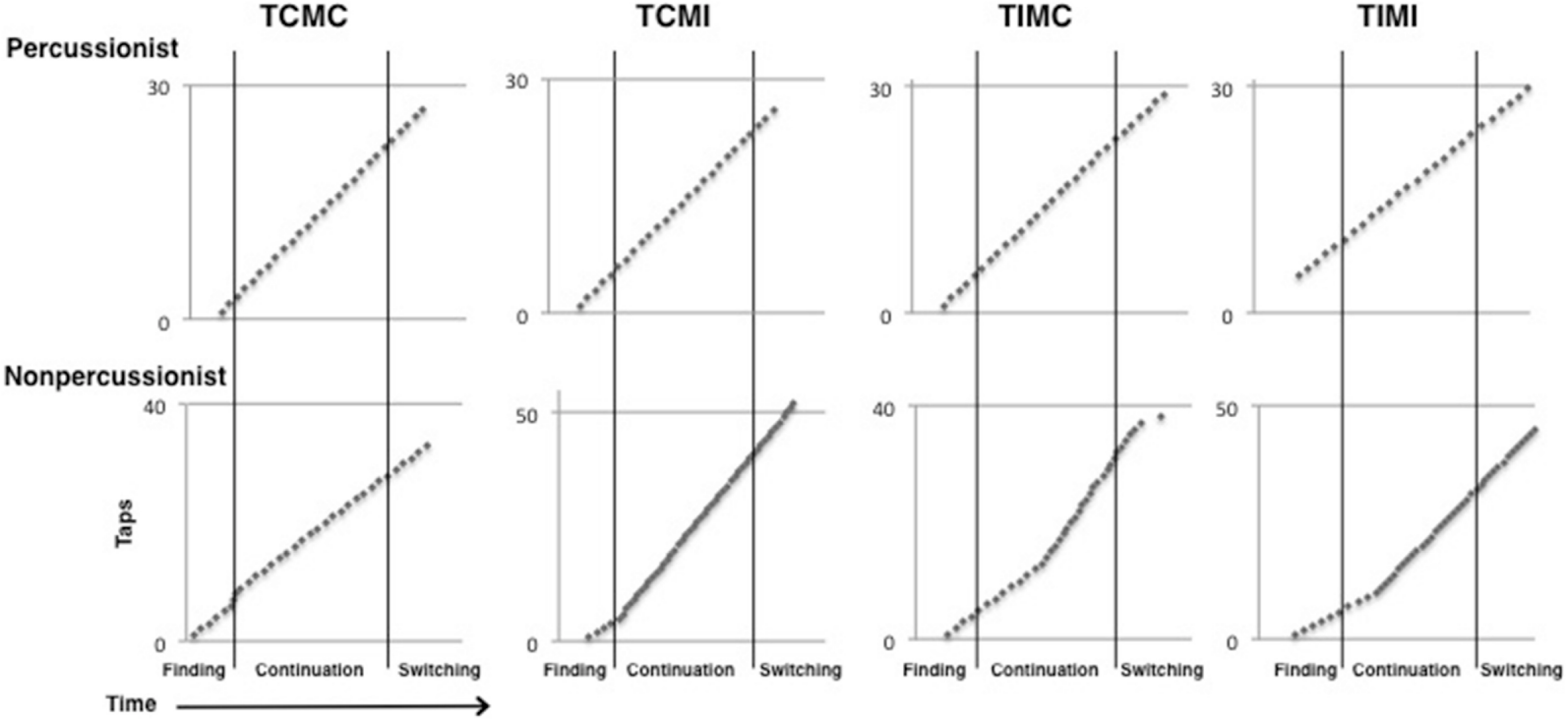
**Beat tapping profiles for each of the four conditions in the beat tapping task from one percussionist and one nonpercussionist participant**. The nonpercussionist's tapping rate fluctuates more than the percussionist's rate in each of the 4 conditions.

As in the case of CV, the negative effect of metrical incongruence on tapping accuracy was greater during switching compared to continuation [interaction of meter congruence and stage: *F*_(1, 50)_ = 4.25, *p* = 0.044]. The negative effect of metrical incongruence on accuracy was also greater for tempo congruent compared to tempo incongruent trials [marginal interaction of metrical and tempo congruence: *F*_(1, 50)_ = 3.25, *p* = 0.077]. Unlike for CV, percussionists' advantage in tapping accuracy was reduced for tempo incongruent trials [interaction of group and tempo congruence: *F*_(1, 50)_ = 12.48, *p* < 0.001]. Furthermore, that interaction was influenced by stage, in a 3 way interaction, such that the degree to which the group difference depended on tempo congruence differed between continuation and switching [*F*_(1, 50)_ = 5.47, *p* = 0.023]. In fact, in tempo incongruent trials, nonpercussionists had greater accuracy than percussionists during the switching stage, and percussionists had greater accuracy during the continuation stage (see Figure 4 for mean results, and Figure 5 for beat tapping profiles from a percussionist and a nonpercussionist).

In order to explore the relationship between percussionists' training and their beat tapping performance, we tested whether each of the 4 measures of percussionists' training (hours of playing percussion per week at time of testing, years of percussion lessons, years of playing percussion, and years of musical training) correlated with mean CV and mean absolute asynchrony of beat tapping. No significant correlations were found (*p* > 0.05, in all cases).

### Rhythm reproduction task

**Figure 6:**
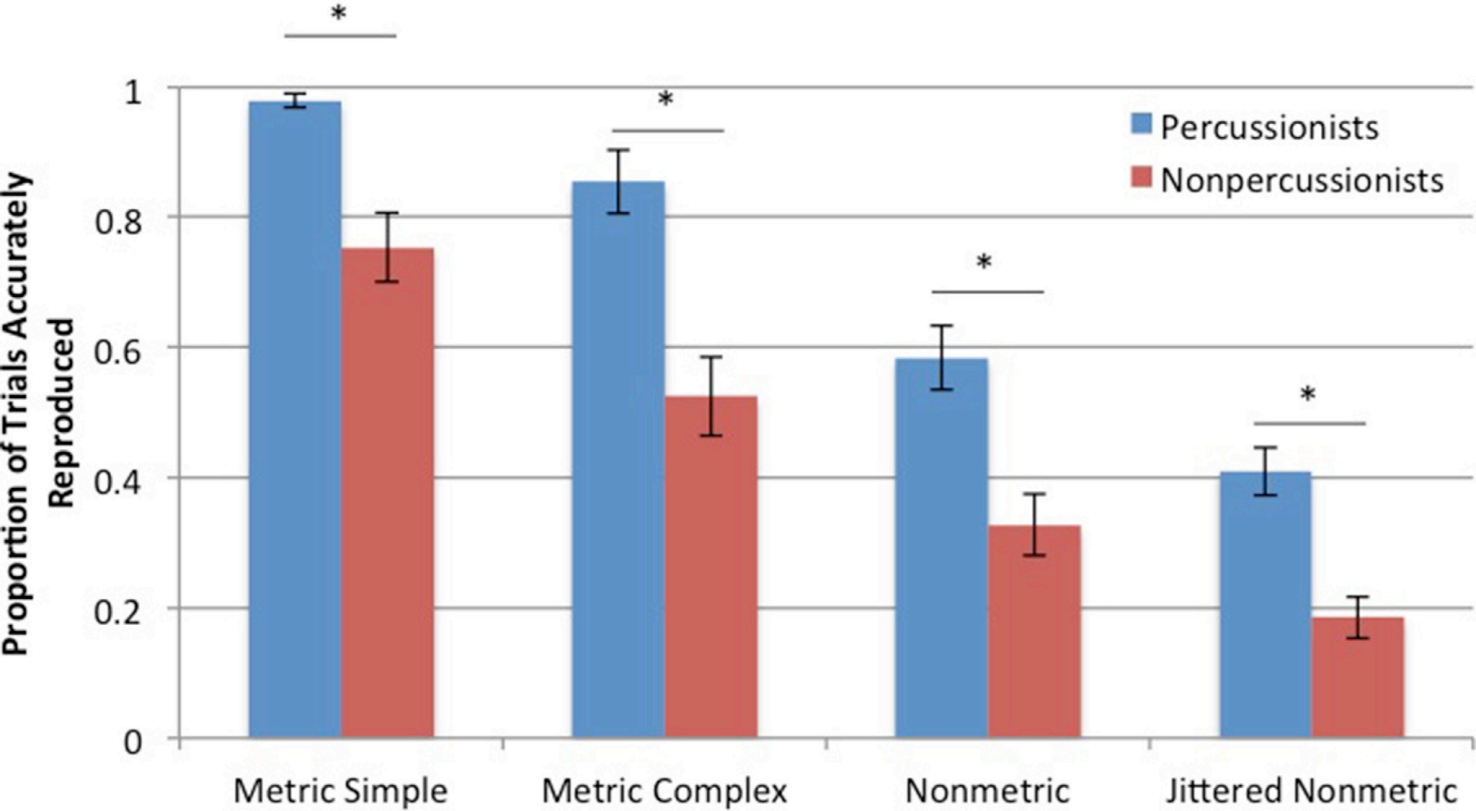
**Mean proportions of accurately reproduced trials in the rhythm reproduction task**. Error bars indicate ± 1 s.e.m. ^*^Indicates *p* < 0.001.

Percussionists accurately reproduced a greater proportion of rhythms compared to nonpercussionists [main effect of group: *F*_(196, 1)_ = 64.88, *p* < 0.001]. Both groups were influenced by rhythm type [main effect of rhythm type: *F*_(196, 1)_ = 64.96, *p* < 0.001]. These two factors did not interact, indicating that the effect of rhythm type did not differ between percussionists and nonpercussionists. The differences in proportion of accurately reproduced rhythms (for both groups) between MS and MC rhythms, between MC and NM rhythms, and between NM and JNM rhythms were each statistically reliable, based on follow up paired *t*-tests (*p* < 0.001 in all cases), as shown in Figure 6.

To explore the relationship between percussionists' training and rhythm reproduction accuracy, we assessed whether each of the 4 training measures (hours of playing percussion per week at time of testing, years of percussion lessons, years of playing percussion, and years of total training) correlated with the overall proportion of accurately reproduced rhythms. No significant correlations were found (*p* > 0.05, in all cases).

## Discussion

### Beat tapping task

#### Tapping variability

Overall, percussionists showed less variable beat tapping compared to nonpercussionists, and this advantage extended to musically implausible (tempo incongruent) contexts. The CV of tapping was lower for percussionists than nonpercussionists in all stages and across all conditions. Both groups demonstrated more variable beat tapping when the two rhythmic sequences had incongruent tempi, as shown in Figure 3, but percussionists' tapping was still less variable than that of nonpercussionists in these conditions. Thus, the findings confirm previous reports of superior beat tapping ability in percussionists (Fischinger, 2009), and indicate that this advantage generalizes beyond the beat-based rhythmic contexts that are found in music. The superior performance is presumably due to extensive training that prioritizes the ability to maintain a steady beat, although we cannot reject the possibility that individuals with superior timing abilities are more attracted to percussion training.

The effect of metrical incongruence on tapping variability was only seen during the switching stage, and depended on tempo congruence. Specifically, during switching, tapping variability was higher in the TCMI condition than in all other conditions (TCMC, TIMC, TIMI). This is likely because in the other conditions the tapping rate either did not need resetting because the beat rate was the same as the initial sequence (TCMC trials), or needed resetting due to tempo incongruence, rendering the metrical congruence irrelevant (TIMC, TIMI trials). In other words, metrical incongruence only caused beat tapping variability to increase when participants switched between two sequences that had been integrated into a single metrical structure due to having a common underlying pulse.

#### Tapping accuracy

Tapping accuracy results were broadly consistent with those of variability, and with our predictions: percussionists' beat tapping was superior to that of nonpercussionists, and tapping was negatively affected by tempo incongruence and by metrical incongruence during the switching stage. Percussionists had more accurate beat tapping (lower mean absolute asynchrony between taps and beat positions) than nonpercussionists during the finding stage, when only a single rhythm was presented. Both groups tapped less accurately for the tempo incongruent than tempo congruent conditions. However, unlike tapping variability, tapping accuracy was worse overall for metrically incongruent trials than metrically congruent trials. This negative effect of metrical incongruence was greater during switching than continuation, likely because during continuation, participants were able to tap with the beat of the ongoing initial sequence, whereas during switching, the initial sequence stopped, requiring participants to switch their tapping to the new beat rate of the remaining sequence. Taps during the switch would naturally have worse accuracy until participants locked in to the new beat rate. The negative influence of metrical incongruence was also greater during tempo congruent trials than tempo incongruent trials, presumably because during tempo incongruent trials, the misalignment of the basic units of each sequence renders the sequences sufficiently unrelated that the congruence of their meters becomes irrelevant. By contrast, during tempo congruent trials, the basic units are aligned but the metrical structure of the second sequence does not align with that of the first, making the overall stimulus more complex (though still musically plausible), and thus more difficult to tap to.

Unlike for tapping variability, percussionists' advantage in tapping accuracy was not uniform across all conditions and stages. During continuation, tempo incongruence caused percussionists' advantage over nonpercussionists to shrink, although percussionists were still better than nonpercussionists. During switching, however, tempo incongruence caused percussionists' advantage to disappear entirely: percussionists actually had *lower* accuracy than nonpercussionists (although this difference was only marginally significant) during the switch stage of tempo incongruent trials. Percussionists' lower accuracy in this condition contrasts with their better scores on tapping variability. It may be that percussionists' superior entrainment to the beat of the initial sequence caused them to continue tapping the beat of that sequence even after it finished (see Figure 5). As their taps would be aligned with the sequence that had finished, not the sequence currently playing, their accuracy would appear reduced but variability would be unchanged. We would expect that percussionists' tapping would be more accurate than nonpercussionists' during the switch stage of tempo incongruent trials if given a longer period to adjust to the new rhythmic sequence after the switch, or if specifically instructed to adapt to the new beat rate as quickly as possible.

The beat tapping task findings indicate that percussionists do indeed have a greater ability to accurately entrain their tapping to the beat of paired rhythmic sequences than nonpercussionists, that beat tapping of both groups is negatively influenced by tempo incongruence, and that percussionists' advantage persists even when the sequences are incongruent in tempo, thus musically implausible.

### Rhythm reproduction task

Percussionists performed better than nonpercussionists when reproducing rhythmic sequences of all types: those with a strong metrical beat (MS), those with a weak metrical beat (MC), those with no metrical beat structure (NM), and those with less inherent predictability (a greater number of distinct interval lengths possible in a rhythm, JNM). In both groups, as rhythmic beat structure and predictability decreased, reproduction performance also decreased (see Figure 6). Thus, both percussionists and nonpercussionists use the regular beat structure of rhythms to improve the accuracy of timed movements, but percussionists' superior temporal production is not dependent on the presence of a beat structure. The NM and JNM conditions are unlikely to be encountered in music, therefore the reproduction results are consistent with the beat tapping results: percussionists' superior abilities extend to nonbeat-based, musically implausible sequences.

Reproduction performance was worse for JNM rhythms compared to NM rhythms. This implies that, at least in the absence of any beat structure, other temporal factors influence perception and reproducibility. One factor suggested by these results is that inherent predictability influences how accurately rhythms are reproduced. Previous work shows that ability to reproduce single intervals is sensitive to the distribution of intervals presented during the task (Jazayeri and Shadlen, 2010; Cicchini et al., 2012). Thus, the larger number of possible temporal intervals in the JNM rhythms may reduce the predictability of the JNM sequences compared to the NM sequences (which contained only 4 possible temporal intervals). Future approaches could use probabilistic modeling and manipulation of the predictability in rhythmic sequences to test how the number of possible intervals in a rhythm affects reproducibility.

### General discussion

Overall percussionists were more accurate and less variable during beat tapping, and better able to accurately reproduce rhythms, regardless of beat structure. Percussionists' superior performance might be due to their training. Percussion training aims to enhance temporal precision of manual actions, particularly in reference to auditory sequences, and emphasizes the ability to maintain a steady and precise beat. However, these data do not demonstrate a causal influence of training directly and we cannot rule out other causal explanations, such as preexisting advantages in temporal processing that would explain both improved performance and likelihood of beginning and maintaining percussion training. In fact, there was no significant correlation between measures of percussionists' training and their task performance. But, because our sample of percussionists was limited (mostly undergraduate percussion students), and had a somewhat narrow range of training experience, this study (as opposed to a longitudinal study, for example) is not well suited to reflect on specific causal influences of percussion training.

Percussionists and nonpercussionists might have used different strategies in these tasks. For example, in the beat tapping task, percussionists may have been able to track both rhythms and their respective beat positions, improving their performance. However, two studies suggest that tracking the beat in two simultaneous rhythms is unlikely to have occurred. When presented with two rhythmic sequences with different beat rates, expert musicians could *not* track both beat rates except in the simplest arrangement (most similar to our tempo congruent meter incongruent condition), possibly by integrating the two into a single, composite rhythm with a single beat rate (Poudrier and Repp, 2013). Such integration is unlikely to be possible in our tempo incongruent conditions. In another study, musicians' ability to detect perturbations in one of two isochronous sequences declined as the complexity of the ratio of the two rates increased (e.g., 7:5 is more complex than 2:5) and as the pitch difference between the two sequences decreased (Fidali et al., 2013). Our tempo incongruent trials were composed of sequences whose beat rates were not integer ratios of one another, and thus were more complex than the most complex conditions in that study, and the two sequences in every trial had the same pitch. Thus, it is highly unlikely that participants tracked the beat of both sequences simultaneously.

Anecdotally, some percussionists reported that some reproduction trials (presumably NM and JNM rhythms) were surprisingly difficult. In addition, when asked if they thought they had performed better than nonpercussionists could have performed in these specific trials, they said no or were not sure. This suggests a lack of awareness of the extent and nature of their expertise. It also suggests that percussionists relied on implicit timing skills, rather than explicit, intentional strategies for these trials.

Taken together, these results suggest that percussionists have superior timing performance, and that their superiority does not rely on a rhythm's beat structure or musical plausibility. Since beat structure in rhythms allows or causes entrainment, these data show that percussionists' expertise with rhythm production includes the ability to maintain entrained tapping to complex, musically implausible rhythmic stimuli (they showed superior beat tapping variability and accuracy in incongruent conditions), but does not rely on entrainment (they showed superior rhythm reproduction accuracy for rhythms without a beat). Together, these results show that both entrainment and absolute (unentrained) interval reproduction are timing skills honed by percussionists. Although these results do not demonstrate a causal effect of training, they are consistent with the possibility that the neural mechanisms underlying both beat-based and nonbeat-based timing (Teki et al., 2011) are tuned over time by percussion training.

### Conflict of interest statement

The authors declare that the research was conducted in the absence of any commercial or financial relationships that could be construed as a potential conflict of interest.
