# Supplementary material for: Enhanced timing abilities in percussionists generalize to rhythms without a musical beat
Source: Front Hum Neurosci. 2014 Dec 10;8:1003. doi: 10.3389/fnhum.2014.01003 (PMC4262051; doi:10.3389/fnhum.2014.01003)
Supplement: Supplementary file 1 [file Table1.PDF]

Supplementary Material

Enhanced Timing Abilities in Percussionists Generalize to Rhythms Without a Musical Beat

Daniel J. Cameron[1]*, Jessica A. Grahn[1,2]
[1]Brain and Mind Institute, Western University, London, Ontario, Canada
[2]Dept. of Psychology, Western University, London, Ontario, Canada
*** Correspondence:** Daniel Cameron, Brain and Mind Institute, Western University, Natural Sciences Centre, London, Ontario, N6A 5B7, Canada.
dcamer25@uwo.ca

Supplementary Table 1. Supplementary audio files are as follows.

Name of .wav file	Description
Audio 1	Example Tempo Congruent Metric Congruent (TCMC) trial from the beat tapping task.
Audio 2	Example Tempo Congruent Metric Incongruent (TCMI) trial from the beat tapping task.
Audio 3	Example Tempo Incongruent Metric Congruent (TIMC) trial from the beat tapping task.
Audio 4	Example Tempo Incongruent Metric Incongruent (TIMI) trial from the beat tapping task.
Audio 5	Example Metric Simple (MS) trial from the reproduction task. One presentation of the stimulus to be reproduced by the participant.
Audio 6	Example Metric Complex (MC) trial from the reproduction task. One presentations of the stimulus to be reproduced by the participant.
Audio 7	Example Nonmetric (NM) trial from the reproduction task. One presentation of the stimulus to be reproduced by the participant.
Audio 8	Example Jittered Nonmetric (JNM) trial from the reproduction task. One presentation of the stimulus to be reproduced by the participant.
